# Comments on ‘Global research trends in tongue cancer from 2000 to 2022: bibliometric and visualized analysis’, by Wu et al.

**DOI:** 10.1007/s00784-025-06415-0

**Published:** 2025-06-04

**Authors:** Yuh-Shan Ho, Nikolaos Christidis

**Affiliations:** 1 CT HO Trend, 3F.-7, No. 1, Fuxing N. Rd., Songshan Dist, Taipei City, 105611 Taiwan; 2 Division of Oral Rehabilitation, Department of Dental Medicine, Karolinska Institutet, Huddinge, SE-14104 Sweden

## Brief summary

Wu et al. recently published a paper in *Clinical Oral Investigations* titled ‘Global research trends in tongue cancer from 2000 to 2022: Bibliometric and visualized analysis [1]. ’ The purpose of this study was to do a bibliometric analysis of the global research trends on tongue cancer performed during the years 2000 and 2022. As the authors stated, this is an important topic to address given the increasing incidence of tongue cancer, driven partly by human papillomavirus (HPV), and the ongoing challenges in clinical management. The specific objectives, as set by the authors were to identify key topics, research trends, and collaborations within the scientific community, thereby providing a clearer understanding of the evolution and key focus areas in tongue cancer research.

Their analysis covered 2205 publications involving contributions from 72 countries, 2233 institutions, and 11,266 authors. They could also show a substantial growth in research production, particularly since 2016. China emerged as the leading country in publication volume, whereas the USA held dominance in citations and impact metrics. Key institutions included the Karolinska Institutet and Memorial Sloan-Kettering Cancer Center. Important research themes identified were apoptosis, depth of invasion, HPV, radiotherapy, and surgical margins. The authors concluded that the research interest in tongue cancer is steadily increasing, highlighting HPV, depth of invasion, tumour budding, and surgical margins as central themes.

## Comment on published paper

Scientific research serves as the cornerstone for uncovering fundamental truths, whereas innovation is essential for developing novel perspectives or enhancing existing knowledge [2]. To achieve meaningful progress, researchers must continuously refine their methodologies and persistently explore relevant questions, rather than revisiting previously studied areas without notable advancement. In 2011, Ho’s team proposed the concept of the “front page,” a filtering tool specifically developed to enhance search strategies within bibliometric research. This tool utilises Topic (TS) terms from the Web of Science Core Collection [3], focusing on essential elements such as titles, abstracts, and author keywords to reduce the inclusion of unrelated studies in bibliometric assessments.

The application of this filtering approach to research topics within the SCI-EXPANDED and SSCI databases revealed considerable variations, highlighting its importance in improving data quality. For instance, using the “front page” filter resulted in deviations of 15% in studies on keloids [4], 14% in research on Q-fever [5], 15% in research on temporomandibular disorders [6], 20% in research addressing managerial aspects of construction and demolition waste [7], and 27% in studies on algal-bacterial symbiosis in wastewater treatment [8]. These discrepancies underscore the critical role played by this filtering mechanism in enhancing bibliometric analyses, ensuring research remains focused and relevant, and thereby enabling more accurate insights and conclusions.

Wu et al. presented the following search strategies in the section of ‘Data sources and collection’ in their paper ‘Global research trends in tongue cancer from 2000 to 2022: Bibliometric and visualized analysis’ [1]:

### Database

Science Citation Index Expanded (SCI-EXPANDED) and Social Sciences Citation Index (SSCI).

### Year published (PY)

2000–2022.

### Document type (DT)

article or review.

### Topic (TS)

“Tongue Neoplasms” OR “Neoplasm, Tongue” OR “Tongue Neoplasm” OR “Neoplasms, Tongue” OR “Cancer of Tongue” OR “tongue cancers” OR “Cancer of the Tongue” OR “tongue cancer” OR “Cancer, Tongue” OR “Cancers, Tongue”.

However, the Web of Science Core Collection includes:

1. Science Citation Index Expanded (SCI-EXPANDED) -- 1900-present.
2. Social Sciences Citation Index (SSCI) -- 1900-present.
3. Arts & Humanities Citation Index (A&HCI) -- 1975-present.
4. Conference Proceedings Citation Index - Science (CPCI-S) -- 1990-present.
5. Conference Proceedings Citation Index - Social Sciences & Humanities (CPCI-SSH) -- 1990-present.
6. Book Citation Index– Science (BKCI-S) -- 2005-present.
7. Book Citation Index– Social Sciences & Humanities (BKCI-SSH) -- 2005-present.
8. Emerging Sources Citation Index (ESCI) -- 2015-present.
9. Current Chemical Reactions (CCR-EXPANDED) -- 1985-present.
10. Index Chemicus (IC) -- 1993-present.

When we employed the search strategy outlined by Wang et al. [1], a total of 2,047 articles and reviews were identified, with 2,024 of these published between 2000 and 2022, and data updated as of 10 March 2025. These publications were sourced from the Social Science Citation Index (SSCI) and the Science Citation Index Expanded (SCI_EXPANDED). However, a discrepancy emerges when comparing this updated dataset (2,024 papers) with the original dataset by Wu et al. [1], which comprised only 1,873 English articles and reviews. This discrepancy equates to an 8.1% variation between the two datasets.

Of the 2,024 articles and reviews examined, only 1,713 (85% of the total) contained search keywords within their title, abstract, or author keywords. This finding implies that a significant portion of the literature may not directly align with the specific focus on tongue cancer research, suggesting possible gaps or inaccuracies within the search strategies applied.

Additionally, Wu et al. [1] overlooked to include several relevant keywords related to tongue cancer, such as “tongue carcinoma,” “tongue carcinomas,” “tongue malignant tumor,” “tongue malignancies,” and “tongue malignancy.” Consequently, numerous pertinent articles and reviews were omitted from the original dataset by Wu et al. [1], leading to an incomplete representation of the tongue cancer research landscape.

In conclusion, the analysis by Wu et al. [1] underscores significant deficiencies in the search strategy that could potentially misinform readers, particularly within *Clinical Oral Investigations*. These deficiencies highlight the critical importance of adopting comprehensive and validated search methodologies in bibliometric research to ensure the accuracy, reliability, and integrity of the outcomes. Future studies must emphasize the development and use of meticulous search strategies to provide credible and valuable contributions to this field.

## Data Availability

No datasets were generated or analysed during the current study.

## References

[1] Wu BB, Zhang T, Dai N, Luo D, Wang XJ, Qiao C, Liu J (2024) Global research trends in tongue cancer from 2000 to 2022: bibliometric and visualized analysis. Clin Oral Invest 28:130. 10.1007/s00784-024-05516-6 10.1007/s00784-024-05516-6 38305810

[2] Ho YS (2019) Some comments on: Mao (2018) Bibliometric analysis of insights into soil remediation *Journal of Soils and Sediments*, 18(7): 2520–2534. J Soils Sediments 19:3657–3658. 10.1007/s11368-019-02322-6

[3] Wang MH, Ho YS (2011) Research articles and publication trends in environmental sciences from 1998 to 2009. Archives Environ Sci 5:1–10

[4] Chong YM, Long X, Ho YS (2021) Scientific landscape and trend analysis of keloid research: A 30-year bibliometric review. Ann Transl Med 9:945. 10.21037/atm-21-508 34350260 10.21037/atm-21-508 PMC8263893

[5] Farooq M, Khan AU, El-Adawy H, Mertens-Scholz K, Khan I, Neubauer H, Ho YS (2022) Research trends and hotspots of Q fever research: A bibliometric analysis 1990–2019. BioMed Res Int 2022:9324471. 10.1155/2022/9324471 35075431 10.1155/2022/9324471 PMC8783702

[6] Al-Moraissi EA, Christidis N, Ho YS (2023) Publication performance and trends in temporomandibular disorders research: A bibliometric analysis. J Stomatol Oral Maxillofac Surg 124:101273. 10.1016/j.jormas.2022.08.016 36057419 10.1016/j.jormas.2022.08.016

[7] Ho YS Comment on, Chen J, Su Y, Si H, Chen J (2019) Managerial Areas of Construction and Demolition Waste: A Scientometric Review. *Int J Environ Res Public Health* 2018, 15, 2350. Int J Environ Res Public Health 16:1837. 10.3390/ijerph16101837 10.3390/ijerph16101837 PMC6572021 31126135

[8] Ho YS (2019) Comment to: Qi, Yi, Bibliometric Analysis of Algal-Bacterial Symbiosis in Wastewater Treatment, *Int. J. Environ. Res. Public Health* 2019, 16, 1077. Int J Environ Res Public Health 16:2034. 10.3390/ijerph16112034 10.3390/ijerph16061077 PMC6466313 30917551

